# Supplementary figures and images for: Genomic discovery of EF-24 targets unveils antitumorigenic mechanisms in leukemia cells
Source: PLoS One. 2025 Sep 23;20(9):e0330906. doi: 10.1371/journal.pone.0330906 (PMC12456773; doi:10.1371/journal.pone.0330906)

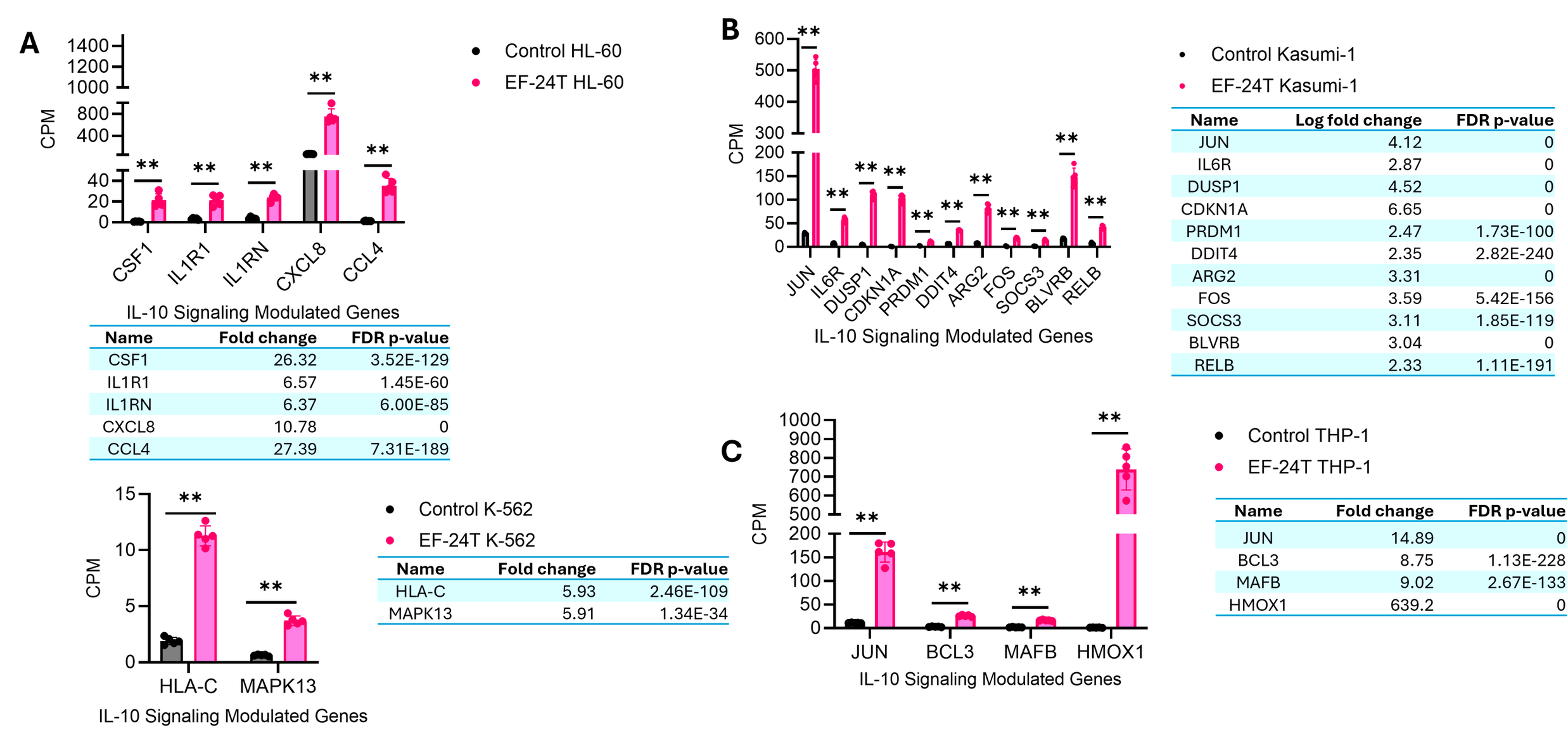

Supplement: S1 Fig — (A–D) Bar plots show the relative mRNA expression levels of the indicated genes in EF-24–treated versus untreated control samples for each cell line representing distinct myeloid leukemia subtypes. False Discovery Rate (FDR) p-value ** < 0.01. (TIF) [file pone.0330906.s001.tif]

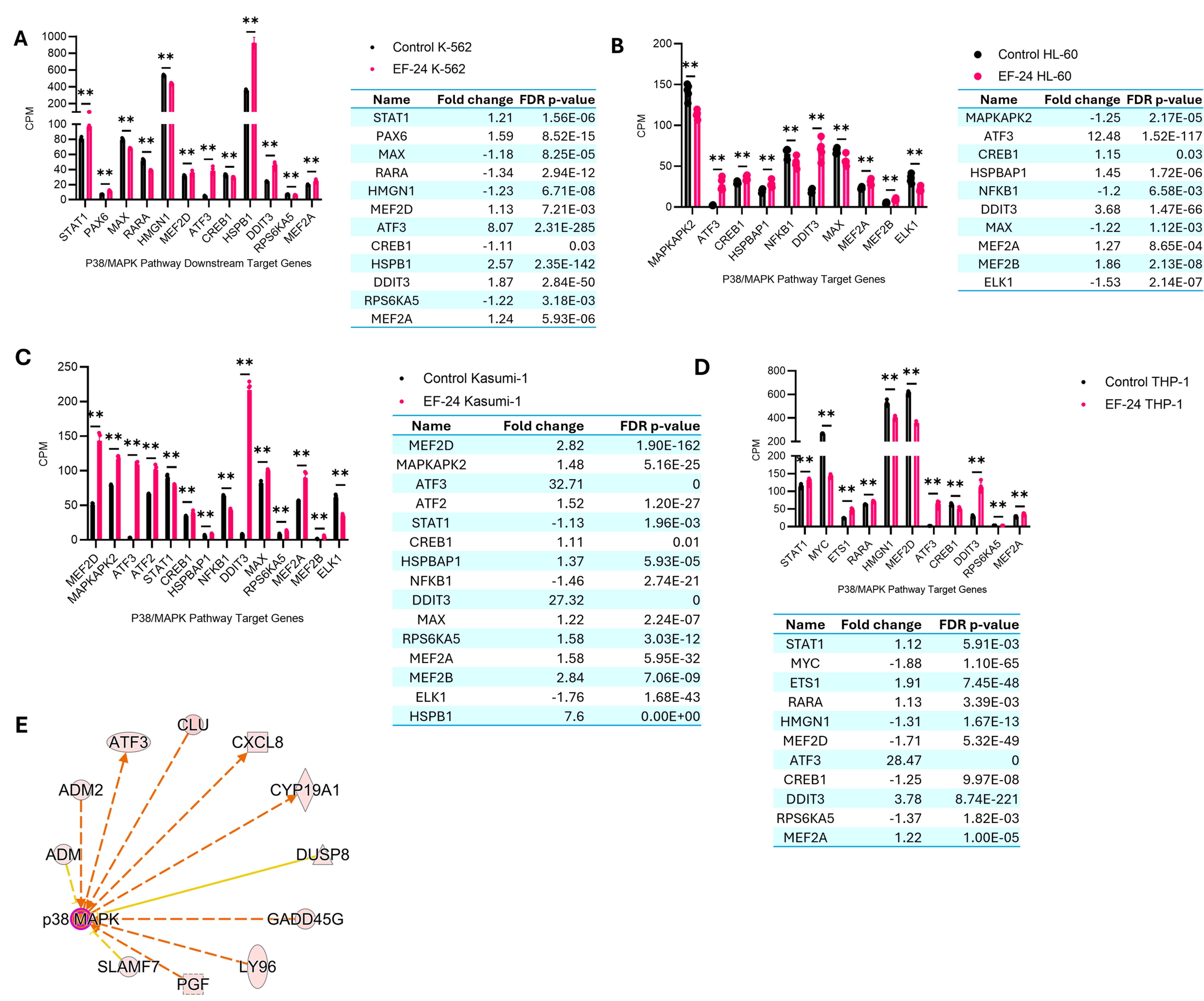

Supplement: S2 Fig — (A–C) Bar plots show the relative mRNA expression levels of the indicated genes in EF-24–treated versus untreated control samples for each cell line representing distinct myeloid leukemia subtypes. False Discovery Rate (FDR) p-value ** < 0.01. (TIF) [file pone.0330906.s002.tif]
