# Supplementary material for: Genomic discovery of EF-24 targets unveils antitumorigenic mechanisms in leukemia cells
Source: PLoS One. 2025 Sep 23;20(9):e0330906. doi: 10.1371/journal.pone.0330906 (PMC12456773; doi:10.1371/journal.pone.0330906)
Supplement: Attachment — Submitted filename: comments.docx [file pone.0330906.s003.docx]

1. The discussion could appropriately mention the dual roles of signaling pathways in cancer.
2. Please standardize terminology; for instance, is the phrase "complete cell death" (line 77) scientifically justified?
3. While the study emphasizes coverage of diverse leukemia subtypes, it fails to justify the exclusion of common subtypes (e.g., FLT3-ITD mutated AML). Please clarify this rationale and discuss the generalizability of conclusions to other subtypes.
