# Supplementary material for: Genomic discovery of EF-24 targets unveils antitumorigenic mechanisms in leukemia cells
Source: PLoS One. 2025 Sep 23;20(9):e0330906. doi: 10.1371/journal.pone.0330906 (PMC12456773; doi:10.1371/journal.pone.0330906)
Supplement: Attachment — Submitted filename: EF24_Singh_review PLOSone.docx [file pone.0330906.s004.docx]

In the manuscript entitled “Genomic Discovery of EF-24 Targets Unveils Antitumorigenic Mechanisms in Leukemia Cells”, Singh, A.P. et al. perform whole transcriptome sequencing on 4 human leukemia cell lines after treatment with the curcumin analog, EF-24. The authors show that there are both conserved and unique gene expression changes upon a 24 hour treatment with the drug. Some of the affected pathways are important in cell viability and inflammatory response, therefore suggesting an antitumorigenic response. This is a useful dataset to understand the acute effects to the EF-24 compound which is still being characterized for its efficacy. However, there is very limited orthogonal validation of many of the DEGs discussed in the manuscript. Moreover, given that most of the manuscript is focused on the results in individual cell lines, it is difficult to discern how applicable these results are for the use of EF-24 in primary leukemia samples.

Major critiques/questions:

1) The authors highlight that EF-24 has shown efficacy in leukemia cells in a prior publication. However, between the prior publication and the manuscript herein, there has been no assays with non-transformed hematopoietic cells. If the authors want to be able to state that the gene expression differences they are seeing are specific to the leukemic cells, they should perform the same whole transcriptome sequencing in CD34+ bone marrow cells to determine how many of the pathways changing are leukemia specific.
2) There has been a previous publication detailing a mechanism for EF-24 in killing leukemia cells. However, that publication focused only on the protein and signaling pathway activation aspect of the response. The data described herein, if analyzed through the lens of the p38/MAPK/ERK pathway target genes, may be a way to strengthen the manuscript and integrate the data from each cell line if they are responding through the upregulation or downregulation of certain pathway members.
3) While many of the genes differentially expressed are notable and described for their potential importance in aiding leukemic cell death, there is very little orthogonal validation outside of replotting their own transcriptome sequencing data. The authors should validate some of the key genes through qPCR or evaluate the overall change to certain pathways via Western. For instance, in Figure 5, the data suggests a central IL-10 signaling signature and mention that this would lead to the upregulation of certain target genes. However, the expression of those genes is not shown even from the transcriptome sequencing data. Further validation of the genes/pathway highlighted for each cell line would add impact to the findings.
4) The authors utilize 3 AML cell lines but only 1 CML cell line. It would be worthwhile to add additional CML cell lines and then perform an analysis examining the differences between lymphoid/myeloid leukemia cell lines to determine if there is a significant divergence in cellular response based on the lineage of the leukemia. May also prioritize some of the pathways already identified as central pathways altered by EF-24 treatment.
5) It is unclear if statistical analysis was performed on any of the data in Figure 1 or statistical values provided for any of the gene expression data highlighted throughout.

Minor critiques/questions:

1) More detailed Figure titles denoting the main finding in the figure would be helpful to orient the reader.
2) Many panels in the Figures are quite hard to read at their current size. It would be helpful to make many of the network maps larger and more legible.
